# Hypersensitivity of Prelimbic Cortex Neurons Contributes to Aggravated Nociceptive Responses in Rats With Experience of Chronic Inflammatory Pain

**DOI:** 10.3389/fnmol.2018.00085

**Published:** 2018-03-22

**Authors:** Xiao-Cen Fan, Su Fu, Feng-Yu Liu, Shuang Cui, Ming Yi, You Wan

**Affiliations:** ^1^Neuroscience Research Institute and Department of Neurobiology, School of Basic Medical Sciences, Peking University, Beijing, China; ^2^Key Laboratory for Neuroscience, Ministry of Education/National Health and Family Planning Commission, Peking University, Beijing, China

**Keywords:** pain, chronic pain, prelimbic cortex, prefrontal cortex, p38

## Abstract

Previous experience of chronic pain causes enhanced responses to upcoming noxious events in both humans and animals, but the underlying mechanisms remain unclear. In the present study, we found that rats with complete Freund’s adjuvant (CFA)-induced chronic inflammatory pain experience exhibited aggravated pain responses to later formalin test. Enhanced neuronal activation upon formalin assaults and increased phosphorylated cAMP-response element binding protein (CREB) were observed in the prelimbic cortex (PL) of rats with chronic inflammatory pain experience, and inhibiting PL neuronal activities reversed the aggravated pain. Inflammatory pain experience induced persistent p38 mitogen-activated protein kinase (MAPK; p38) but not extracellular regulated protein kinase (ERK) or c-Jun N-terminal kinase (JNK) hyperphosphorylation in the PL. Inhibiting the p38 phosphorylation in PL reversed the aggravated nociceptive responses to formalin test and down-regulated enhanced phosphorylated CREB in the PL. Chemogenetics identified PL–periaqueductal gray (PAG) but not PL–nucleus accumbens (NAc) as a key pathway in inducing the aggravated formalin pain. Our results demonstrate that persistent hyperphosphorylation of p38 in the PL underlies aggravated nociceptive responses in rats with chronic inflammatory pain experience.

## Introduction

Chronic pain is one of the most prevalent clinical situations. The experience of chronic pain affects physiological states of the individual, even after the pain has perceptually recovered. A subject with chronic pain experience frequently shows enhanced responses to following noxious events, reflected in lower pain thresholds and increased pain ratings (Bachiocco et al., 1993; Lidow, 2002; Ren et al., 2004; Hermann et al., 2006; Wegner et al., 2015). In animal studies, enhanced formalin-evoked pain behaviors are also observed in adult rats with chronic inflammatory pain experience (Li et al., 2012). However, mechanisms underlying deteriorated pain responses following previous pain experience are not well illustrated.

Medial prefrontal cortex (mPFC) shows long-term morphological and functional changes in chronic pain (Apkarian et al., 2004; Metz et al., 2009; Seminowicz et al., 2009; Lim et al., 2016), which parallel long-term behavioral patterns such as anxiety, stress and depression (Negrón-Oyarzo et al., 2014; Fitzgerald et al., 2015; Moench and Wellman, 2015; Seo et al., 2017). Even when the chronic pain has been well treated, the neuroanatomical abnormality could only be partly reversible (Seminowicz et al., 2011). Prelimbic cortex (PL) is part of the rodent mPFC and plays a critical role in perceptual and emotional aspects of chronic pain (Baliki et al., 2012; Wang et al., 2015; Wu et al., 2016). Excitability of layer 4/5 pyramidal neurons in the PL increases in complete Freund’s adjuvant (CFA)-induced inflammatory mice (Wu et al., 2016), and bilateral lesions of the PL attenuate CFA-induced heat hyperalgesia (Wang et al., 2015). PL may regulate pain through its projections to a number of other brain areas (Kucyi et al., 2013; Khan et al., 2014; Yu et al., 2014; Lee et al., 2015; Vachon-Presseau et al., 2016), including amygdala, nucleus accumbens (NAc), hippocampus and in particular periaqueductal gray (PAG), a critical component of the descending pain modulatory system (Umana et al., 2017).

Several molecular signaling pathways underlie the altered neuronal activities in the PL in chronic pain. The mitogen-activated protein kinase (MAPK), including extracellular regulated protein kinase (ERK), p38 MAPK (p38) and c-Jun N-terminal kinase (JNK), is a family of serine/threonine protein kinases that transduces extracellular stimuli into intracellular post-translational and transcriptional responses (Seger and Krebs, 1995; Widmann et al., 1999; Johnson and Lapadat, 2002; Obata and Noguchi, 2004). Besides its role in chronic stress, depression and fear conditioning (Sherrin et al., 2011; Ferland et al., 2014; Pochwat et al., 2017), the MAPK also contributes to pain hypersensitivity by regulating neuronal plasticity and inflammatory responses in dorsal root ganglion (DRG), spinal cord and cerebral cortex (Impey et al., 1999; Ji and Woolf, 2001; Kumar et al., 2003; Gao and Ji, 2008; Ji et al., 2009). Chronic pain is accompanied by hyperphosphorylation of MAPKs in both peripheral and central nervous system (Zhuang et al., 2005; Crown et al., 2006, 2008; Cao et al., 2014), whereas inhibition of MAPKs impairs neuronal excitability and relieves pain (Hu and Gereau, 2003; Wynne, 2006; Toyoda et al., 2007; Zhang et al., 2007; Pucilowska et al., 2012).

Based on the findings above, we hypothesize that persistent functional alterations in the PL facilitate nociceptive responses to subsequent noxious exposure, even after the perceptual recovery of chronic pain. To test this hypothesis, we examined molecular changes of the PL in rats with experience of CFA-induced chronic inflammatory pain, and observed sustained PL hyper-reactivity which mediated the vulnerability to formalin pain test in these rats.

## Materials and Methods

### Animals

Adult male Sprague-Dawley rats (230–250 g at the beginning of experiments) were provided by the Department of Laboratory Animal Sciences, Peking University Health Science Center (Beijing, China). All animals were housed in standard cages with a 12-h alternating light/dark cycle and food and water available *ad libitum*. All experimental procedures were approved by the Animal Care and Use Committee of our University, according to the guidelines of the International Association for the Study of Pain. By the end of the experiment, euthanasia was performed with 1% pentobarbital sodium (1 ml/100 g, *i.p*.).

### Establishment of CFA-Induced Inflammatory Pain Model of Rats

Following our previous protocol (Yue et al., 2017; Zhang C. et al., 2017), the rat was anesthetized with isoflurane. The plantar surface of left hindpaw was cleaned by 75% ethanol before a total of 100 μL CFA was injected intraplantarly. For control, an equal volume of normal saline was injected.

### Measurement of Thermal and Mechanical Pain Thresholds

The rat was handled for 10 min, and adapted in a plexiglas box for 30 min per day for three consecutive days before the first measurement. Thermal or mechanical pain thresholds were measured as previously described (Chaplan et al., 1994; Zhang et al., 2016) while the rat was calm and awake. Paw withdrawal latencies (PWLs) to thermal stimuli were measured by a focused radiant heat (40 W of power) applied to either hindpaw (Hargreaves Method, IITC 390). PWLs were recorded three times and averaged as the thermal pain threshold. A cut-off value of 30 s was set to avoid any possible tissue injuries.

Fifty percent paw withdrawal thresholds (50% PWTs) to mechanical stimuli were measured by *von* Frey hairs (0.41–15.1 g; North Coast, Gilroy, CA, USA). The *von* Frey hair was applied to the central plantar surface of either hindpaw. The 50% PWTs were calculated by the “up and down” method as described by Chaplan et al. and in our lab (Chaplan et al., 1994; Zhang et al., 2016). Thermal hyperalgesia and mechanical allodynia were measured 1 day before and 1, 3, 7, 14, 21 and 28 days after CFA injection.

### Hot Plate Test

The rat was handled for 10 min and adapted in the hot plate for 10 min per day for three consecutive days before the test. Rats were placed individually onto the center of the hot plate (49°C) and the latency of the first sign of hind paw licking or jumping to avoid heating pain was recorded as an index of the nociceptive threshold (Luo et al., 2004; Yu et al., 2008). A cut-off value of 30 s was set to avoid any possible tissue injuries. The hot plate was cleaned by 75% ethanol between tests.

### Open Field Test

The rat was placed in a 100 × 100 × 50 cm box exposed to 50 lux illumination, with its activities videotaped for 10 min (Zhang M. et al., 2017; Zhang Y. et al., 2017). Time spent (C.Time) and distance traveled (C.Dis) in the central area (60 × 60 cm), and total distance traveled (T.Dis) in the field were measured using the SMART software (v2.5.21, Panlab, Harvard Apparatus). The box was cleaned by 75% ethanol between tests.

### Elevated Plus-Maze Test

The elevated plus-maze test was carried out on the next day of the open field test (Li et al., 2017; Zhang M. et al., 2017). The maze was placed 50 cm above the floor in a 30 lux illuminated room and consisted of two open arms and two closed arms (48 × 8 cm, and 40 cm wall height for the closed arms). The rat was placed onto the center area, heading toward the same open arm, and videotaped in the following 10 min. Time spent (O.Time) and numbers of entries (O.Entries) into open arms and total arm entries (T.Entries) were analyzed. The maze was cleaned by 75% ethanol between tests.

### Formalin Test

Formalin test was performed 30 days after the CFA injection. Rats were handled for 10 min and adapted in a plexiglas chamber for 20 min per day for 3 days before test. The rat received an injection of 100 μL of 5% standard formalin solution into the plantar surface of right hindpaw (the opposite hindpaw of CFA/saline injection), with its behavior videotaped in the following 60 min. Time spent on licking and lifting the formalin injected paw were counted, and the formalin pain score was calculated as previously described: (time lifting + 2 × time licking)/total time (Yi et al., 2011; Zhang et al., 2014). The chamber was cleaned by 75% ethanol between tests.

Brains were removed 45 min after formalin injection for Western blotting and immunostaining, or 90 min after formalin injection for c-Fos immunostaining.

To test anxiety-like behaviors of rats after the formalin injection, open field and elevated plus-maze tests were performed 1 and 2 days after formalin injection, respectively.

### Capsaicin Test

Capsaicin test was performed 30 days after the CFA injection. The plantar surface of right hindpaw was cleaned by 75% ethanol before a total of 5 μg (0.1 μg/μl) capsaicin was injected intraplantarly (Chen et al., 2006). Evoked nociceptive responses were measured by focused radiant heat (as described above) 15, 30, 60, 90 and 120 min after capsaicin injection (Soliman et al., 2005).

### Cannula Implantation and Drug Microinjection

The rat was anesthetized deeply with 1% sodium pentobarbital (0.5 ml/100 g, *i.p*.) and positioned in a stereotaxic frame (RWD, Shenzhen, China). A guide cannula (O.D. 0.48 mm/I.D. 0.34 mm, C.C 1.2 mm, RWD, Shenzhen, China) was implanted 1.5 mm above PL [anterior-posterior (AP) + 2.9 mm; medial lateral (ML) ± 0.6 mm from Bregma; dorsal-ventral (DV) −2.5 mm from brain surface] (Zhang et al., 2016). Four skull screws were used for securing the guide cannula to the skull surface with dental acrylic. The matching cap (0.5 mm below the guide cannula, RWD, Shenzhen, China) was inserted into the guide cannula. All animals were given at least 1 week for recovery from surgery before further experiments. The injection needle (1.5 mm below the guide cannula, RWD, Shenzhen, China) was used for microinjection with a polyethylene catheter connecting a micro-syringe. GABA_A_R agonist muscimol (1 μg/μl, 0.5 μl/side, Tocris Bioscience), p38 inhibitor SB203580 (1 μg/μl, 0.5 μl/side, Sigma-Aldrich) or vehicle (artificial cerebrospinal fluid, aCSF, 0.5 μl/side) was injected into PL of either side over 2 min. The injection needle was held on for at least 2 min to allow drug diffusion. The behavioral tests were performed 30 min after drug/vehicle injection. Rats with incorrect site of the guide cannula were excluded from analysis.

### Stereotaxic Microinjection of Adeno- Associated Virus (AAV) Vectors Into PL

AAV5-CaMKIIα-hM4D(Gi)-mCherry and AAV5-CaMKIIα-mCherry viruses were packaged and purchased from the University of North Carolina Vector Core Facilities (Zhang Y. et al., 2017). AAV virus solution was microinjected into PL (AP +2.7/3.2 mm; ML ± 0.6 mm from Bregma; DV −2.5 mm from brain surface) with 0.5 μl/hole, 2 holes/side, at a speed of 0.1 μl/min after being anesthetized with 1% sodium pentobarbital (0.5 ml/100 g, *i.p*.). The needle was kept on the site for 3 min to allow for virus diffusion and gradually withdrawn over 1 min to prevent possible leakage from the needle track. The behavioral tests were performed 6 weeks after virus injection.

### Stereotaxic Microinjection of Clozapine-N-Oxide (CNO)

For local clozapine-N-Oxide (CNO) delivery, a guide cannula (O.D. 0.48 mm/I.D. 0.34 mm, C.C 1.6 mm, RWD, Shenzhen, China) was implanted 2 mm above ventrolateral periaqueductal gray matter (vlPAG; AP −7.8 mm; ML ± 0.8 mm from Bregma; DV −6.0 mm from brain surface), and guide cannula (O.D. 0.48 mm/I.D. 0.34 mm, C.C 3, 0 mm, RWD, Shenzhen, China) was implanted 2 mm above NAc core (AP +1.5 mm; ML ± 1.5 mm from Bregma; DV −7.7 mm from brain surface). CNO (1 mmol/L, 0.5 μl/side, Tocris Bioscience) was injected into vlPAG or NAc core slowly. Behavioral tests were performed 30 min after the CNO injection.

### Western Blotting

The rat brain was removed, embedded in optimum cutting temperature compound (catalog #0201 08926, Leica), and frozen in liquid nitrogen immediately. PL tissues were taken out by using No.9 puncture needles in a cryostat microtome according to the stereological location (Zheng et al., 2017). Protein was extracted by RIPA (C1053, Applygen Technologies Inc.) and mixed with a 6× loading buffer (DE0105, Beijing BioDee BioTech Corporation Ltd.). Thirty microgram protein in each hole was separated in 10% SDS-PAGE gel and transferred onto polyvinylidene fluoride membrane (ISEQ00010, Merck Millipore). The membrane was blocked with 5% bull serum albumin at room temperature for 1 h, then incubated with rabbit anti-p38 antibody (1:1000, 8690, Cell Signaling Technology), or anti-p-p38 antibody (1:1000, 4511, Cell Signaling Technology), or anti-cAMP-response element binding protein (CREB) antibody (1:1000, 9197, Cell Signaling Technology), or anti-p-CREB antibody (1:1000, 9198, Cell Signaling Technology), or anti-ERK antibody (1:1000, 4695, Cell Signaling Technology), or anti-p-ERK antibody (1:1000, 4370, Cell Signaling Technology), or anti-JNK antibody (1:1000, 9252, Cell Signaling Technology), anti-p-JNK antibody (1:1000, 9251, Cell Signaling Technology), anti-β-tubulin antibody (1:2000, 2128, Cell Signaling Technology) or anti-GAPDH (1:5000, 2118, Cell Signaling Technology) at 4°C for 20 h, washed in Tris-buffered saline and Tween-20 (TBST), and then incubated with horseradish peroxidase-conjugated goat anti-rabbit IgG antibody (1:2000, 111–035–003, Jackson ImmunoResearch) at room temperature for 1 h. Protein bands were detected using Western blotting luminol reagent (sc-2048, Santa Cruz). Data were analyzed with the ImageJ software.

### Immunostaining

The rat was anesthetized with 1% pentobarbital sodium and intracardially perfused with 4% paraformaldehyde (PFA, in 0.1 M phosphate buffer, pH 7.4). The brain was post-fixed with 4% PFA for 12 h, and cryoprotected in 20% and 30% sucrose solutions in turn. 35 μm sections were sliced coronally using a cryostat microtome (Model 1950, Leica Instrument Co., Ltd.) throughout the entire PL (Zheng et al., 2017). Free-floating sections were washed in the phosphate buffered saline (PBS), blocked with a buffer containing 3% bull serum albumin and 0.3% triton X-100 for 1 h, and incubated with the following primary antibodies in 4°C for 24 h: rabbit anti-c-Fos antibody (1:300, sc-52, Santa Cruz), or rabbit anti-p-p38 antibody (1:500, 4511, Cell Signaling Technology), or goat anti-glutamate transporter (EAAC1) antibody (1:1000, AB1520, Merck Millipore), or mouse anti-GFAP antibody (1:1000, 3670, Cell Signaling Technology), or goat anti-Iba1 antibody (1:800, ab5076, Abcam) or mouse anti-GAD67 antibody (1:1000, ab26116, Abcam). Sections were then washed in PBS and incubated with secondary antibodies at room temperature for 90 min: Alexa Fluor 594-conjugated donkey anti-rabbit IgG (1:300, A21207, Invitrogen), or Alexa Fluor 488-conjugated donkey anti-goat IgG (1:300, A11055, Invitrogen) or Alexa Fluor 488-conjugated goat anti-mouse IgG (1:300, ab150113, Abcam). For p-p38 and glutamate transporter staining, sections were incubated in EDTA antigen retrieval solution at 98°C for 30 min to expose epitopes in DNA before blocked with serum. For co-staining of p-p38 and Iba1, antigen retrieval was not applied to avoid poor coloration of Iba1. Images were taken by a laser scanning confocal microscope (model FV1000, Olympus Co., Ltd.).

### Statistical Analysis

Data were presented as means ± SEM. Unpaired or paired two-tailed* t* tests and one-way analysis of variance (ANOVA) with Bonferroni *post hoc* tests were used for the comparison of two groups. Comparisons of two groups with different time points were performed using two-way ANOVA or ANOVA with repeated measures and Bonferroni *post hoc* test. The differences were calculated with software GraphPad Prism 5.0 and statistical significance was defined as *p* < 0.05.

## Results

### Aggravated Nociceptive Responses in Rats With CFA Chronic Pain Experience

**Figure 1:**
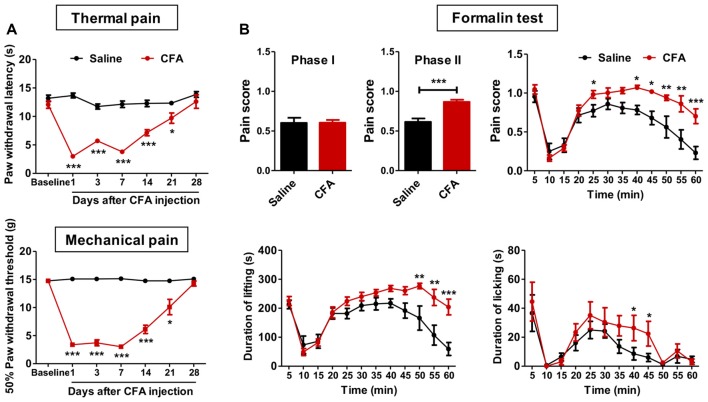
Aggravated formalin-induced pain in rats with chronic inflammatory pain experience. **(A)** Thermal hyperalgesia (top) and mechanical allodynia (bottom) in complete freund’s adjuvant (CFA)-induced chronic inflammatory pain recovered to the baseline level 28 days after CFA injection.* n* = 8 in each group. *p < 0.05, ***p < 0.001, CFA vs. Saline, analyses of variance (ANOVA) with repeated measures and Bonferroni *post hoc* test. **(B)** Increased pain scores in the phase II of the formalin test (top) in rats with chronic inflammatory pain experience. The elevated pain score involved increased durations of both paw lifting behavior (bottom, left) and paw licking behavior (bottom, right). *n* = 8 in each group. *p < 0.05, **p < 0.01, ***p < 0.001, CFA vs. Saline, *t* test, ANOVA with repeated measures and Bonferroni *post hoc* test.

To examine whether chronic pain experience would affect upcoming nociceptive assaults, we first established chronic inflammatory pain with intraplantar CFA injection. These animals showed significant thermal hyperalgesia (group effect: *F*_(1,90)_ = 130.1, *p* < 0.001; time effect: *F*_(6,90)_ = 29.27, *p* < 0.001; interaction: *F*_(6,90)_ = 21.77, *p* < 0.001; Figure 1A) and mechanical allodynia (group effect: *F*_(1,96)_ = 273.6, *p* < 0.001; time effect: *F*_(6,96)_ = 65.37, *p* < 0.001; interaction: *F*_(6,96)_ = 70.02, *p* < 0.001; Figure 1A), which recovered to the baseline level 28 days after CFA injection. PWLs and PWTs of the contralateral paw remained unchanged throughout this period (data not shown). Inflammatory rats exhibited significant paw swelling after CFA injection (on day 1: 3613.0 ± 100.6 vs. 1587.0 ± 60.9 mm^3^; on day 28: 2601.0 ± 49.8 vs. 2042.0 ± 56.9 mm^3^; CFA vs. Saline group).

Anxiety is a common co-morbidity of chronic pain (Zheng et al., 2017). To examine anxiety-like behaviors, we performed open field and elevated plus-maze tests on days 29 and 31 after CFA or saline injection, respectively. We did not observe significant differences between CFA and saline groups at this time point (open field: C.Time, *t*_(16)_ = 0.38, *p* > 0.05; C.Dis, *t*_(16)_ = 0.14, *p* > 0.05; T.Dis, *t*_(16)_ = 1.92, *p* > 0.05; elevated plus-maze: O.Time, *t*_(16)_ = 0.59, *p* > 0.05; O.Entries, *t*_(16)_ = 0.14, *p* > 0.05; T.Entries, *t*_(16)_ = 0.63, *p* > 0.05; Supplementary Figures S1A,B). These results indicate full recovery of thermal hyperalgesia, mechanical allodynia and anxiety-like behaviors in CFA-induced chronic inflammatory pain 28 days after CFA injection.

We next examined these animals’ responses to formalin and capsaicin tests. Rats with CFA pain experience showed significantly higher pain scores in the formalin test (phase I: *t*_(15)_ = 0.04, *p* > 0.05; phase II: *t*_(15)_ = 5.00, *p* < 0.001; Figure 1B), as revealed by increased duration of lifting time and licking time (lifting: group effect: *F*_(1,165)_ = 12.03, *p* < 0.01; time effect: *F*_(11,165)_ = 20.67, *p* < 0.001; interaction: *F*_(11,165)_ = 4.10, *p* < 0.001; licking: group effect: *F*_(1,165)_ = 1.79, *p* > 0.05; time effect: *F*_(11,165)_ = 9.80, *p* < 0.001; interaction: *F*_(11,165)_ = 0.77, *p* > 0.05; Figure 1B). Similarly, aggravated thermal hyperalgesia in the capsaicin test was also observed in rats with chronic pain experience (group effect: *F*_(5,70)_ = 27.56, *p* < 0.01; baseline: *t*_(14)_ = 0.03, *p* > 0.05; 15 min: *t*_(14)_ = 2.56, *p* < 0.05; 30 min: *t*_(14)_ = 2.65, *p* < 0.05; 60 min: *t*_(14)_ = 3.40, *p* < 0.01; 90 min: *t*_(14)_ = 5.10, *p* < 0.001; 120 min: *t*_(14)_ = 3.53, *p* < 0.01; Supplementary Figure S2). However, no differences of anxiety-like behaviors were observed after formalin injection between CFA and saline treated rats (elevated plus-maze: O.Time, *t*_(13)_ = 0.72, *p* > 0.05; O.Entries, *t*_(13)_ = 0.23, *p* > 0.05; T.Entries, *t*_(13)_ = 0.10, *p* > 0.05; open field: T.Dis, *t*_(13)_ = 0.69, *p* > 0.05; Supplementary Figure S3).

Together, these results indicate aggravated nociceptive responses to following noxious assaults in rats with prior chronic pain experience. Given the similar findings from formalin and capsaicin tests, only the formalin test was performed in further experiments.

### Enhanced Activation of PL Neurons Upon Formalin Assaults in Rats With Chronic Pain Experience

**Figure 2:**
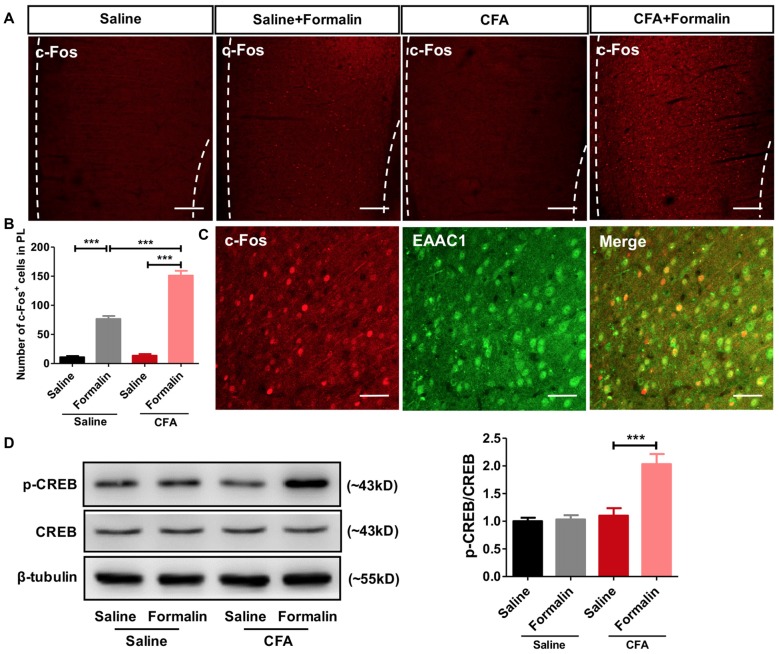
Enhanced activation of prelimbic cortex (PL) neurons to formalin pain in rats with chronic pain experience. **(A)** Representative immunofluorescence images showing c-Fos^+^ neurons in the PL of rats in CFA and saline groups, with or without formalin injection. Scale bars: 200 μm. **(B)** Quantitative analysis of images in **(A)**. Formalin injection induced significant c-Fos expression in the PL, especially in rats with chronic pain experience. *n* = 6 in each group. ***p < 0.001, CFA+Formalin vs. Saline+Formalin, CFA vs. CFA+Formalin, Saline vs. Saline+Formalin, one-way ANOVA. **(C)** The majority (93.70 ± 1.53%) of c-Fos^+^ neurons (red) in PL in CFA+formalin group co-labeled with EAAC1^+^ neurons (green). Scale bars: 50 μm. **(D)** Increased PL p-CREB with formalin injection after chronic pain recovery. Chronic pain experience or formalin injection alone did not affect p-CREB expression in PL in rats. Representative Western blots of p-CREB, CREB and β-tubulin were shown above the corresponding histogram. *n* = 6 in each group. ***p < 0.001, CFA+Formalin vs. CFA+Saline, one-way ANOVA.

To examine whether PL participated in the observed aggravated nociceptive responses, we first performed c-Fos protein mapping after formalin injection. After saline injection, PL showed little c-Fos protein expression in rats with or without chronic pain experience. By contrast, formalin injection induced significant c-Fos expression in PL, especially in rats with chronic pain experience (*F*_(3,85)_ = 159.5, *p* < 0.001; Figures 2A,B). The majority (93.70 ± 1.53%) of c-Fos positive neurons in PL co-labeled with EAAC1, a marker of excitatory glutamatergic neurons (Figure 2C).

Meanwhile, we observed significantly up-regulated phosphorylation of CREB, a marker of neuronal activation, in PL of rats receiving formalin injection with chronic pain experience. However, formalin alone was not sufficient to elevate the content of p-CREB in the PL (*F*_(3,26)_ = 15.45, *p* < 0.001; Figure 2D). These findings indicate stronger activation of PL by formalin pain in rats with chronic pain experience than in those without.

### Inhibiting PL Reverses Aggravated Formalin Pain in Rats With Chronic Pain Experience

**Figure 3:**
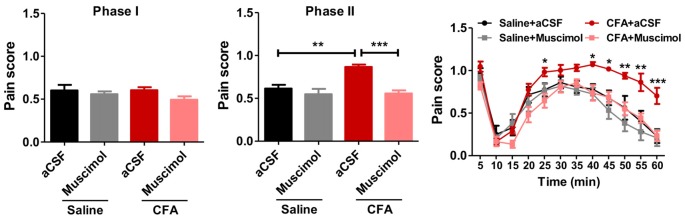
Inhibiting PL reverses aggravated formalin pain in rats with chronic pain experience. Inhibiting PL activities by muscimol relieved the aggravated phase II formalin pain in CFA (the middle column), but not saline (the right column) group. No significant differences in phase I of formalin pain were observed among groups (the left column). *n* = 8 in each group. In the middle column: ***p < 0.001, CFA+aCSF vs. CFA+Muscimol. **p < 0.01, CFA+aCSF vs. Saline+aCSF, one-way ANOVA. In the right column: *p < 0.05, **p < 0.01, ***p < 0.001, CFA+aCSF vs. CFA+Muscimol, ANOVA with repeated measures and Bonferroni *post hoc* test.

To examine whether PL contributed to aggravated nociceptive responses, we injected muscimol, a GABA_A_ receptor agonist, into PL before the formalin test. Muscimol significantly relieved formalin-induced pain behaviors in rats with chronic pain experience, but not in those without (phase I: *F*_(3,33)_ = 1.35, *p* > 0.05; phase II:* F*_(3,33)_ = 11.62, *p* < 0.001; right: group effect: *F*_(3,330)_ = 11.32, *p* < 0.001; time effect: *F*_(11,330)_ = 55.07, *p* < 0.001; interaction: *F*_(33,330)_ = 2.47, *p* < 0.001; Figure 3). These results indicate that PL contributes to aggravated nociceptive formalin pain responses in rats with chronic inflammatory pain experience.

### Persistent Hyperphosphorylation of p38 Accompanies Aggravated Formalin Pain in Rats With Chronic Pain Experience

**Figure 4:**
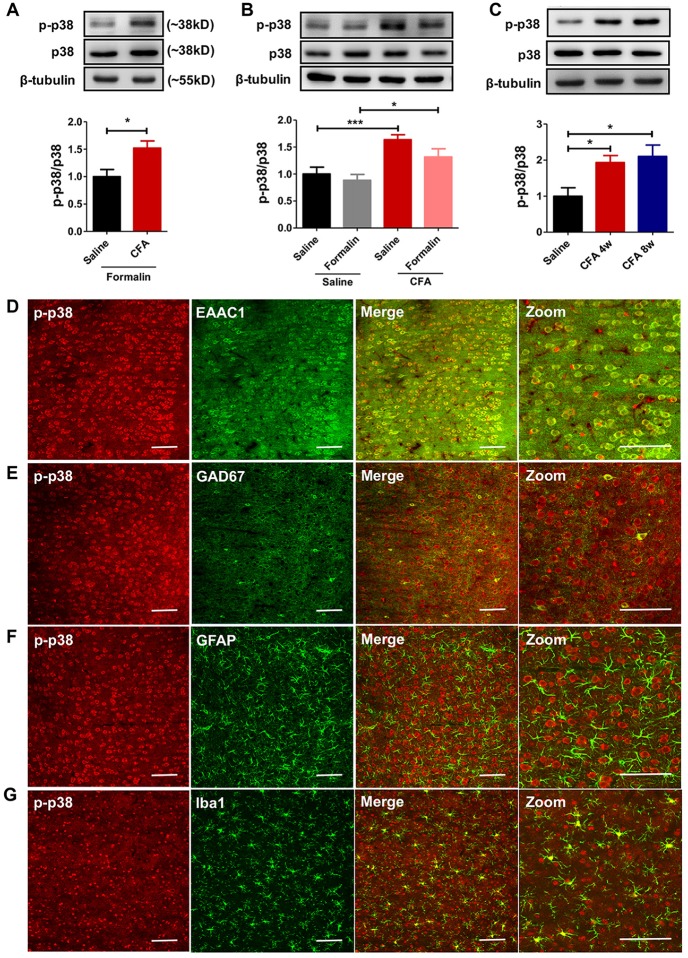
Persistent hyperphosphorylation of p38 accompanies aggravated formalin pain in rats with chronic pain experience. **(A)** Increased PL p-p38 after formalin injection in rats with chronic pain experience. Representative Western blots of p-p38, p38 and β-tubulin were shown above the corresponding histogram.* n* = 7 in each group. *p < 0.05, CFA+Formalin vs. Saline+Formalin, *t* test. **(B)** p-p38 in the PL increased in rats with chronic pain experience, with or without formalin injection. Formalin injection alone did not obviously influence the expression of p-p38 in PL. Representative Western blots of p-p38, p38 and β-tubulin were shown above the corresponding histogram. *n* = 8 in each group. ***p < 0.001, CFA vs. Saline, *p < 0.05, CFA+Formalin vs. Saline+Formalin, one-way ANOVA. **(C)** Hyperphosphorylation of p38 in PL maintained for at least 8 weeks after CFA injection. Representative Western blots of p-p38, p38 and β-tubulin were shown above the corresponding histogram. *n* = 6 in each group. *p < 0.05 CFA 1 m/CFA 2 m vs. Saline, one-way ANOVA. **(D)** Representative immunofluorescence images showing that the majority (87.86 ± 0.84%) of p-p38^+^ neurons (red) co-labeled with EAAC1^+^ neurons (green, a marker of glutamatergic neurons). Scale bars: 100 μm. **(E)** Representative immunofluorescence images showing that few (2.56 ± 0.51%) p-p38^+^ neurons (red) co-labeled with GAD67^+^ neurons (green, a marker of gabaergic neurons). Scale bars: 100 μm. **(F,G)** Representative immunofluorescence images showing that a small proportion of p-p38^+^ neurons (red) co-labeled with GFAP^+^ (1.23 ± 0.16%, green, a marker of astrocytes) and Iba1^+^ cells (8.35 ± 0.66%, green, a marker of microglia). Scale bars: 100 μm.

To explore molecular mechanisms of PL’s contribution to aggravated pain, we examined protein contents of MAPKs (including ERK, p38 and JNK) and their phosphorylation in bilateral PL. MAPKs are key players in nociceptive sensitization, which are activated by various second-messenger signal transduction cascades (Edelmayer et al., 2014). Western blotting showed significantly up-regulated phosphorylation of p38 in PL of rats with chronic pain experience after formalin injection (*t*_(12)_ = 2.80, *p* < 0.05; Figure 4A). Indeed, the up-regulated phosphorylation of p38 was apparent in the PL of rats with chronic pain even without formalin injection (*F*_(3,34)_ = 7.57, *p* < 0.001; Figure 4B). The up-regulated p-p38 was apparent 7 days after CFA injection (*F*_(6,34)_ = 6.25, *p* < 0.001; Supplementary Figure S4), and lasted minimally 8 weeks (*F*_(2,17)_ = 5.51, *p* < 0.05; Figure 4C). By contrast, no significant differences in the content of ERK, p-ERK, JNK or p-JNK were detected (p-ERK/ERK: *F*_(3,33)_ = 1.24, *p* > 0.05; p-JNK/JNK: *F*_(3,37)_ = 1.23, *p* > 0.05; Supplementary Figures S5A,B). Immunofluorescence showed that the majority (87.86 ± 0.84%) of p-p38 positive neurons in PL co-labeled with EAAC1 (Figure 4D), and only a small proportion (2.56 ± 0.51%) with GAD67, a marker of interneurons (Figure 4E). Few astrocytes or microglial cells (GFAP^+^ and Iba1^+^ cells, 1.23 ± 0.16% and 8.35 ± 0.66%, respectively) showed co-expression with p-p38 (Figures 4F,G). These findings indicate that previous chronic pain experience induces sustained hyperphosphorylation of p38 after pain recovery.

### Inhibiting p38 Phosphorylation Reverses Aggravated Formalin Pain in Rats With Chronic Pain Experience

**Figure 5:**
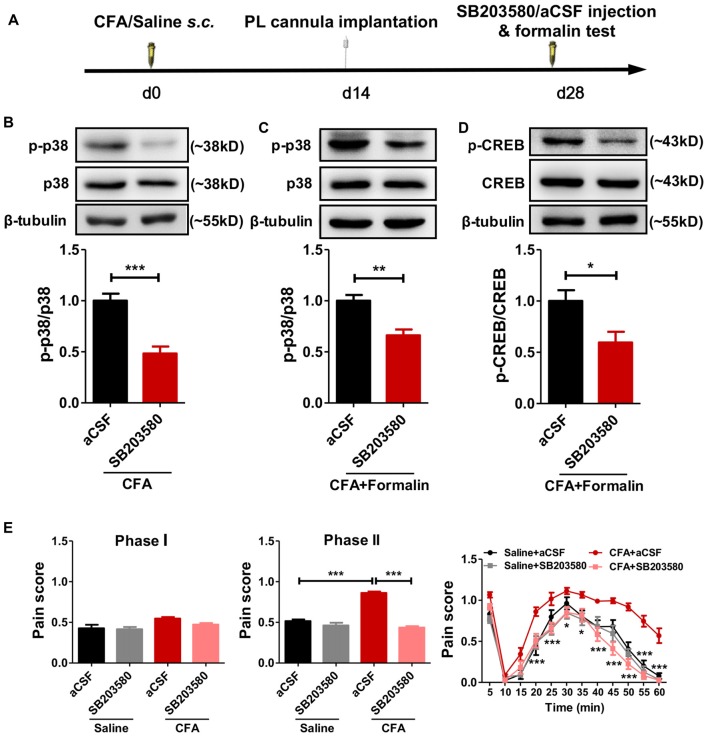
Inhibiting the phosphorylation of p38 reverses aggravated formalin pain in rats with chronic pain experience. **(A)** A diagram showing the experiment time line. SB203580/aCSF micro-injection and formalin test were performed 28 d after CFA/saline injection. The cannula implantation surgery was performed 2 weeks before behavior tests. **(B,C)** SB203580 micro-injection into PL inhibited the hyperphosphorylation of p38 in rats with chronic pain experience, without **(B)** or with formalin injection **(C)**. Representative Western blots of p-p38, p38 and β-tubulin were shown above the corresponding histogram. *n* = 6 in each group. ***p < 0.001, CFA+aCSF vs. CFA+SB203580. **p < 0.01, CFA+aCSF+Formalin vs. CFA+SB203580+Formalin, *t* test. **(D)** SB203580 micro-injection into PL inhibited the hyperphosphorylation of cAMP-response element binding protein (CREB) after formalin injection in rats with chronic pain experience. Representative Western blots of p-CREB, CREB and β-tubulin were shown above the corresponding histogram. *n* = 6 in each group. *p < 0.05, CFA+aCSF+Formalin vs. CFA+SB203580+Formalin, *t* test. **(E)** Inhibiting the phosphorylation of p38 in PL by SB203580 reversed the aggravated phases II formalin pain in the CFA group, but not the saline group (the middle column). Detailed pain scores shown in every 5 min (the right column). *n* = 6 in each group. In the middle column: ***p < 0.001, Saline + aCSF/CFA+SB203580 vs. CFA+aCSF, one-way ANOVA. In the right column: *p < 0.05, ***p < 0.001, CFA+aCSF vs. CFA+SB203580, ANOVA with repeated measures and Bonferroni *post hoc* test.

To examine whether the hyperphosphorylated p38 contributed to sensitized pain behaviors in rats with chronic pain experience, we micro-injected SB203580, a specific inhibitor of p38 phosphorylation, into bilateral PL 30 min before the formalin test (Figure 5A). SB203580 inhibited the hyperphosphorylation of p38 after chronic pain recovery (left: *t*_(10)_ = 5.27, *p* < 0.001; right: *t*_(10)_ = 4.17, *p* < 0.01; Figures 5B,C) and down-regulated formalin-induced p-CREB (*t*_(6)_ = 2.72, *p* < 0.05; Figure 5D).

Behaviorally, SB203580 reversed the aggravated formalin pain in rats with chronic pain experience, but not in those without (phase I: *F*_(3,25)_ = 4.19, *p* < 0.05; phase II:* F*_(3,28)_ = 83.37, *p* < 0.001; right: group effect: *F*_(3,275)_ = 96.95, *p* < 0.001; time effect: *F*_(11,275)_ = 95.58, *p* < 0.001; interaction: *F*_(33,275)_ = 2.64, *p* < 0.001; Figure 5E).

These results suggest that persistent hyperphosphorylation of p38 in the PL contributes to the hypersensitized formalin pain in rats with chronic pain experience.

### PL–PAG Pathway Mediates Aggravated Formalin Pain in Rats With Chronic Pain Experience

**Figure 6:**
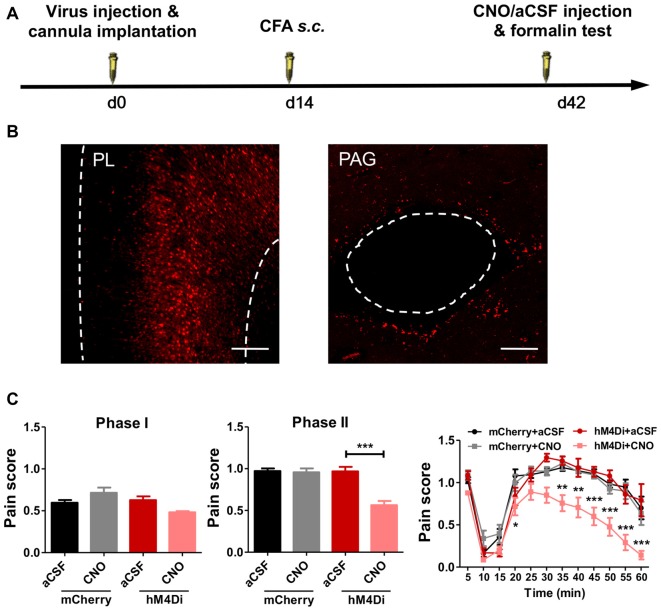
PL–periaqueductal gray (PAG) pathway mediates aggravated formalin pain in rats with chronic pain experience. **(A)** A diagram showing the experiment time line. The clozapine-N-Oxide (CNO)/aCSF micro-injection and formalin test were performed 42 days after virus injection and cannula implantation surgery. CFA injection was performed 28 days before formalin test. **(B)** AAV5-CaMKIIα-hM4D(Gi)-mCherry virus expression in PL (red, left), expression of hM4Di in PL projection in PAG (red, right). Scale bars: 200 μm. **(C)** Compared to the control, inhibiting PL–PAG pathway relieved the aggravated phases II formalin pain in the CFA group (the middle column). Detailed pain scores were shown in every 5 min (the right column). *n* = 8 in each group. In the middle column: ***p < 0.001, hM4Di+aCSF vs. hM4Di+CNO, one-way ANOVA. In the right column: *p < 0.05, **p < 0.01, ***p < 0.001, hM4Di+aCSF vs. hM4Di+CNO, ANOVA with repeated measures and Bonferroni *post hoc* test.

PL projects to several brain areas that modulate pain. We further investigated the neural pathways that aggravated formalin pain in rats with chronic pain experience (Figure 6A). We chemogenetically inhibited the PL–PAG pathway (Figure 6B), which is essential for descending pain modulation, and observed an relieved effect on the aggravated formalin pain in rats with chronic pain experience (phase I: *F*_(3,31)_ = 5.07, *p* < 0.01; phase II:* F*_(3,31)_ = 19.05, *p* < 0.001; right: group effect: *F*_(3,308)_ = 19.04, *p* < 0.001; time effect: *F*_(11,308)_ = 74.70, *p* < 0.001; interaction: *F*_(33,308)_ = 2.23, *p* < 0.001; Figure 6C). By contrast, inhibiting the PL–PAG pathway did not affect physiological pain in rats with chronic pain experience (mCherry: *t*_(21)_ = 1.11, *p* > 0.05; hM4Di: *t*_(5)_ = 1.75, *p* > 0.05; Supplementary Figures S6A,B), nor the development of CFA induced chronic inflammatory pain (*p* > 0.05; Supplementary Figures S7A,B).

In contrast, chemogenetic inhibition of the PL–NAc core pathway did not relieve the aggravated formalin pain in rats with chronic pain experience (phase I: *F*_(3,27)_ = 0.81, *p* > 0.05; phase II:* F*_(3,28)_ = 0.52, *p* > 0.05; right: group effect: *F*_(3,275)_ = 0.16, *p* > 0.05; time effect: *F*_(11,275)_ = 76.15, *p* < 0.001; interaction: *F*_(33,275)_ = 1.27, *p* > 0.05; Supplementary Figures S8A,B).

These findings indicate that PL–mediated aggravated formalin pain in rats with chronic pain experience is achieved through the PL–PAG pathway, but not the PL–NAc core pathway.

## Discussion

### PL Contributes to Aggravated Nociceptive Formalin Pain Responses in Rats With Chronic Inflammatory Pain Experience

Prior pain experience alters future responses to painful stimuli in both human and animals (Bachiocco et al., 1993; Lidow, 2002; Ren et al., 2004; Hermann et al., 2006; Li et al., 2012; Wegner et al., 2015). In the present study, we identified PL as a key region in regulating the aggravated formalin-induced pain in rats after the recovery from CFA-induced chronic inflammatory pain. Rats with chronic pain experience showed elevated pain scores in the second, but not the first, phase of formalin pain. The second phase of formalin pain reflects the development of inflammation and central sensitization, and indicates the involvement of the central nervous system in the aggravated pain (Shibata et al., 1989; Vaccarino and Melzack, 1992).

Structural and functional changes in several brain regions persist long after pain recovery. For example, reduced gray matter densities in chronic back pain patients are observed in the middle cingulate gyrus, thalamus and prefrontal cortex (Ivo et al., 2013), and an increase of gray matter density is found in PAG, thalamus and cerebellum months after whiplash injury and after the headache subsided in most patients with posttraumatic headache (Obermann et al., 2009). Chronic back pain also alters the human brain chemistry: reduction of *N*-acetyl aspartate and glucose is demonstrated in the dorsolateral prefrontal cortex, but not cingulate, sensorimotor and other brain regions (Grachev et al., 2000). In animals, long-term neuropathic pain decreases frontal cortex volumes several months after nerve injury (Seminowicz et al., 2009), and increases basal dendrites of neurons and spine densities in the PL (Metz et al., 2009). These structural and functional changes are accompanied by altered synaptic plasticity and neuronal excitability (Metz et al., 2009; Baliki et al., 2014). Taken together, PL is a brain region particularly vulnerable to chronic pain, regardless of pain recovery or not (Grachev et al., 2000; Apkarian et al., 2004). Some evidences show that pain-induced functional and structural abnormalities in the PFC are at least partially reversible by effective pain treatment (Rodriguez-Raecke et al., 2009; Seminowicz et al., 2011). However, we demonstrated persistent hypersensitivity of PL neurons in rats with chronic pain experience to following pain assaults.

However, we do not consider PL to be the only brain region that contributes to the aggravated nociceptive responses in rats with experience of chronic inflammatory pain. Other areas such as anterior cingulate cortex (ACC) and infralimbic cortex (IL), insular cortex and thalamus are densely connected with PL and undergo plastic changes in chronic pain (Obermann et al., 2009; Ivo et al., 2013; Lin, 2014; Zhuo, 2016; Yue et al., 2017).

### Persistent Hyperphosphorylation of p38 Contributes to Aggravated Formalin Pain in Rats With Chronic Pain Experience

p38 is one of the major MAPK members crucial for generating pain hypersensitivity through transcription-dependent and -independent means (Ji and Woolf, 2001; Crown et al., 2006; Wynne, 2006; Toyoda et al., 2007; Ji et al., 2009). In both peripheral and central nervous system, such as spinal cord and ACC, p38 is activated following pain stimulations, and the activation lasts for more than 3 weeks after chronic constriction injury or spinal nerve ligation (Toyoda et al., 2007; Crown et al., 2008; Cao et al., 2014). Inhibiting p38 in spinal cord or ACC alleviates both subacute and chronic pain (Kumar et al., 2003; Crown et al., 2008; Cao et al., 2014). In the present study, p-p38^+^ neurons are mostly glutamatergic neurons in PL. Under persistent noxious stimulation, hyperphosphorylated p38 can be invoked to activate the downstream signal pathway, including p-CREB and c-Fos, and the activation of nociceptive neurons further sensitizes pain. Inhibiting the phosphorylation of p38 relieves the aggravated formalin pain in rats with chronic pain experience and down-regulates the phosphorylation of its downstream effector CREB and the neuronal activity. The persistent hyperphosphorylation of p38 in PL in rats with chronic pain experience does not directly contribute to pain responses unless a strong stimulus such as formalin injection is performed. These findings indicate that some endogenous inhibitors overlay sensitized p-p38 to keep the balanced “recovery” state.

One candidate of such inhibitors would be layer V pyramidal neurons in the IL, which directly innervate inhibitory interneurons in the PL (Saffari et al., 2016). In addition to pain (Baliki et al., 2012; Wang et al., 2015; Wu et al., 2016), PL also regulates other long-term cognitive behaviors including fear conditioning, working memory, drug addiction and chronic stress (Gisquet-Verrier and Delatour, 2006; Lasseter et al., 2010; Negrón-Oyarzo et al., 2014; Fitzgerald et al., 2015; Moench and Wellman, 2015; Seo et al., 2017). PL neuronal activities maintain freezing behaviors in fear conditioning, whereas extinction induces a novel inhibitory learning in the IL, which antagonize PL pyramidal neuronal activities through local interneurons (Ji and Neugebauer, 2012). These facts lead to an intriguing proposal that the recovery from chronic pain represents pain extinction, instead of simple oblivion of the pain memory (Apkarian et al., 2009; Yi and Zhang, 2011).

### PL–PAG Pathway Mediates Aggravated Formalin Pain in Rats With Chronic Pain Experience

PL has direct projections to several nuclei, including ACC, NAc, amygdala and PAG. Previous studies have revealed roles of PL–NAc and PL–PAG pathways in regulating chronic pain (Kucyi et al., 2013; Yu et al., 2014; Lee et al., 2015). Activation of the PL to NAc core circuit inhibits persistent neuropathic pain and relieves the affective symptoms associated with chronic pain (Lee et al., 2015). Functional and structural connectivity between PAG and mPFC relates to individual differences in attention to pain (Kucyi et al., 2013). PAG is a critical component of the descending pain modulatory system, and usually exerts an inhibitory effect on nociceptive transmission (Umana et al., 2017). Evidence shows that glutamatergic projections from mPFC act to inhibit PAG function (Franklin et al., 2017). Pharmacogenetic inhibition of the projections from PL to PAG therefore disinhibits PAG, and in turn relieves the aggravated formalin-induced pain. The finding that paw lifting behaviors in response to formalin injection, indicating peripheral reflexes, increase in rat with chronic pain experience also supports the involvement of the descending pathway.

Different from the role of PAG in perceptual part of pain, NAc is more involved in pain affection, such as depression-like behaviors in the chronic neuropathic pain state (Goffer et al., 2013; LeBlanc et al., 2015; Navratilova et al., 2015; Kaneko et al., 2017). The present study shows that PL–PAG, but not PL–NAc, pathway regulates the aggravated formalin pain in rats with chronic pain experience, which reflects mostly aggravated pain sensation.

### PL as a Potential Therapeutic Target

Patients with chronic pain have less PFC deactivation than controls during cognition or visual attention tasks (Baliki et al., 2008; Seminowicz et al., 2011), in keeping with our results that the PL is more strongly activated during the following noxious assault in rats with chronic pain experience. Previous work has identified the PL as a target for pain treatment. Repetitive transcranial magnetic stimulation, deep brain stimulation, cognitive-behavioral therapy or other therapies can lead abnormal function of prefrontal cortex to stable or normal state (Fierro et al., 2010; Seminowicz et al., 2013; Boccard et al., 2015), thus relieves the aggravated nociceptive responses in patients with chronic pain experience.

In conclusion, our present study demonstrates that persistent hyperphosphorylation of p38 in the PL underlies aggravated nociceptive responses in rats with chronic inflammatory pain experience, and indicates inhibiting the activity of PL as a promising option for treating hyperalgesia in patients with chronic pain experience.

## Author Contributions

X-CF, MY and YW designed experiments and wrote the manuscript; X-CF, SF, F-YL and SC performed the experiments and analyzed the data; YW and MY supervised the experiments.

## Conflict of Interest Statement

The authors declare that the research was conducted in the absence of any commercial or financial relationships that could be construed as a potential conflict of interest.

## Supplementary Material

FIGURE S1 Similar levels of anxiety-like behaviors in chronic pain rats 30 days after CFA injection. **(A)** Rats showed similar levels of anxiety-like behaviors in the open field test 30 days after CFA injection, indicated by similar time spent (C.Time, left) and distance traveled (C.Dis, middle) in the central area of the open field between groups. Total distance traveled (T.Dis, right) in the field were not affected either. *n* = 8 in each group. CFA vs. Saline, *t* test. **(B)** Rats showed similar levels of anxiety-like behaviors in the elevated plus-maze test 30 days after CFA injection, indicated by similar time spent (O.Time, left) and entries (O.Entries, middle) into the open arms between groups. Total arm entries (T.Entries, right) were not affected either. *n* = 8 in each group. CFA vs. Saline, *t* test.

FIGURE S2 Aggravated capsaicin-induced pain in rats with chronic inflammatory pain experience. Rats with chronic inflammatory pain experience showed increased thermal hyperalgesia 15, 30, 60, 90 and 120 min after capsaicin injection. *n* = 8 in each group. *p* < 0.01, Saline+Capsaicin vs. CFA+Capsaicin, one-way ANOVA; **p* < 0.05, ***p* < 0.01, ****p* < 0.001, Saline+Capsaicin vs. CFA+Capsaicin, *t* test.

FIGURE S3 Similar levels of anxiety-like behaviors after formalin injection between CFA chronic pain group and saline control group. **(A)** Rats with chronic pain experience showed similar levels of anxiety-like behaviors in the elevated plus-maze test after formalin injection, indicated by similar time spent (O.Time, left) and entries (O.Entries, middle) into the open arms between groups. Total arm entries (T.Entries, right) were similar either. *n* = 8 in each group. CFA+Formalin vs. Saline+Formalin, *t* test. **(B)** Similar levels of locomotion after formalin injection between CFA and saline groups, indicated by similar total distance traveled (T.Dis) in the field. *n* = 8 in each group. CFA+Formalin vs. Saline+Formalin, *t* test.

FIGURE S4 Increased levels of p-p38 in the PL after CFA injection. p-p38 in the PL increased in chronic pain rats 7 days after CFA injection, and maintained for at least 28 days. Representative Western blots of p-p38, p38 and β-tubulin were shown above the corresponding histogram. *n* = 5 in each group. ****p* < 0.001, ***p* < 0.01, vs. Saline, one-way ANOVA.

FIGURE S5 Chronic pain experience and formalin injection do not affect the phosphorylation of ERK1/2 and JNK in PL. **(A)** Similar levels of p-ERK1/2 in PL in rats with chronic pain experience. Formalin injection did not affect the phosphorylation of ERK1/2 in PL in rats with or without chronic pain experience. Representative Western blots of p-ERK1/2, ERK1/2 and β-tubulin were shown above the corresponding histogram. *n* = 8 in each group, one-way ANOVA. **(B)** Lack of significant changes of p-JNK in PL in rats with chronic pain experience. Formalin injection did not affect the phosphorylation of JNK in PL in rats with or without chronic pain experience. Representative Western blots of p-JNK, JNK and GAPDH were shown above the corresponding histogram. *n* = 8 in each group, one-way ANOVA.

FIGURE S6 Inhibiting PL–PAG pathway does not affect physiological pain after chronic inflammatory pain recovery. **(A)** A diagram showing the time line of the experiment. The CNO/aCSF micro-injection and hot plate test were performed 28 days after CFA injection. Virus injection and cannula immplantation surgery were performed 14 days before CFA injection. **(B)** Compared to the control, inhibiting PL–PAG pathway did not affect physiological pain after chronic inflammatory pain recovery. *n* = 6 in each group. Before vs. After CNO injection, paired *t* test.

FIGURE S7 Inhibiting PL–PAG pathway does not affect thermal hyperalgesia in the development of CFA-induced chronic inflammatory pain. **(A)** A diagram showing the experiment time line. CNO/aCSF micro-injection and thermal hyperalgesia test were performed 1 day before and 1, 3, 7, 14, 21 and 28 days after CFA injection. Virus injection and cannula implantation surgery were performed 6 weeks before CFA injection. **(B)** Compared to the control, inhibiting PL–PAG pathway did not influence thermal hyperalgesia in the development of CFA-induced chronic inflammatory pain. *n* = 7 in each group. Before vs. After CNO injection, paired *t* test.

FIGURE S8 PL–NAc core pathway does not affect aggravated formalin pain in rats with chronic pain experience. **(A)** Confirmation of AAV5-CaMKIIα-hM4D(Gi)-mCherry virus expression in PL projection in NAc core (red). Scale bars: 100 μm. **(B)** Inhibiting PL–NAc core pathway did not relieve the aggravated formalin pain in either phase I (the left column) or phase II (the middle column) in CFA group. *n* = 8 in each group, one-way ANOVA. Detailed pain scores were shown in every 5 min (the right column). *n* = 8 in each group, ANOVA with repeated measures and Bonferroni *post hoc* test.
