# Supplementary figures and images for: Hypersensitivity of Prelimbic Cortex Neurons Contributes to Aggravated Nociceptive Responses in Rats With Experience of Chronic Inflammatory Pain
Source: Front Mol Neurosci. 2018 Mar 22;11:85. doi: 10.3389/fnmol.2018.00085 (PMC5874315; doi:10.3389/fnmol.2018.00085)

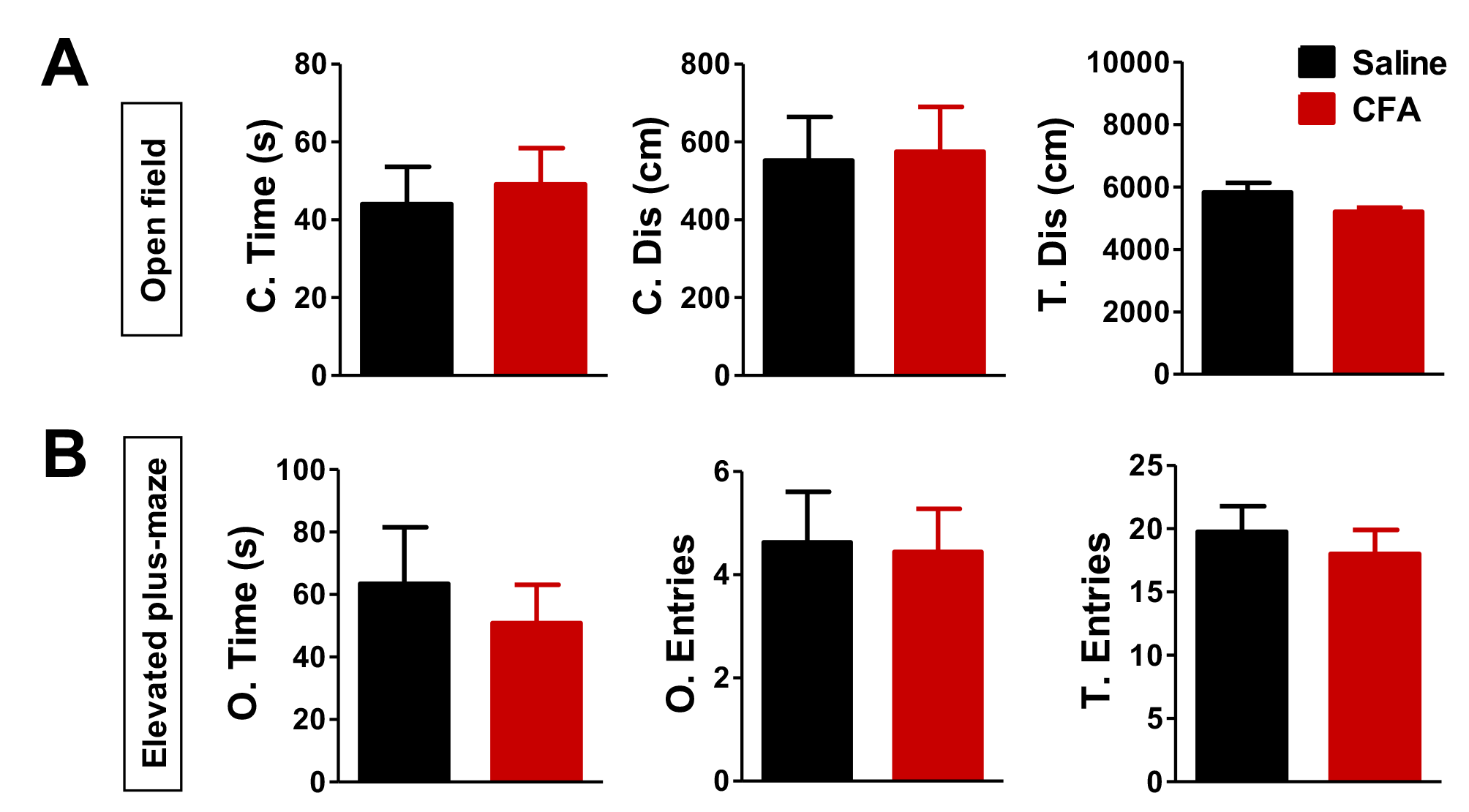

Supplement: FIGURE S1 — Similar levels of anxiety-like behaviors in chronic pain rats 30 days after CFA injection. (A) Rats showed similar levels of anxiety-like behaviors in the open field test 30 days after CFA injection, indicated by similar time spent (C.Time, left) and distance traveled (C.Dis, middle) in the central area of the open field between groups. Total distance traveled (T.Dis, right) in the field were not affected either. n = 8 in each group. CFA vs. Saline, t test. (B) Rats showed similar levels of anxiety-like behaviors in the elevated plus-maze test 30 days after CFA injection, indicated by similar time spent (O.Time, left) and entries (O.Entries, middle) into the open arms between groups. Total arm entries (T.Entries, right) were not affected either. n = 8 in each group. CFA vs. Saline, t test. [file Image_1.TIF]

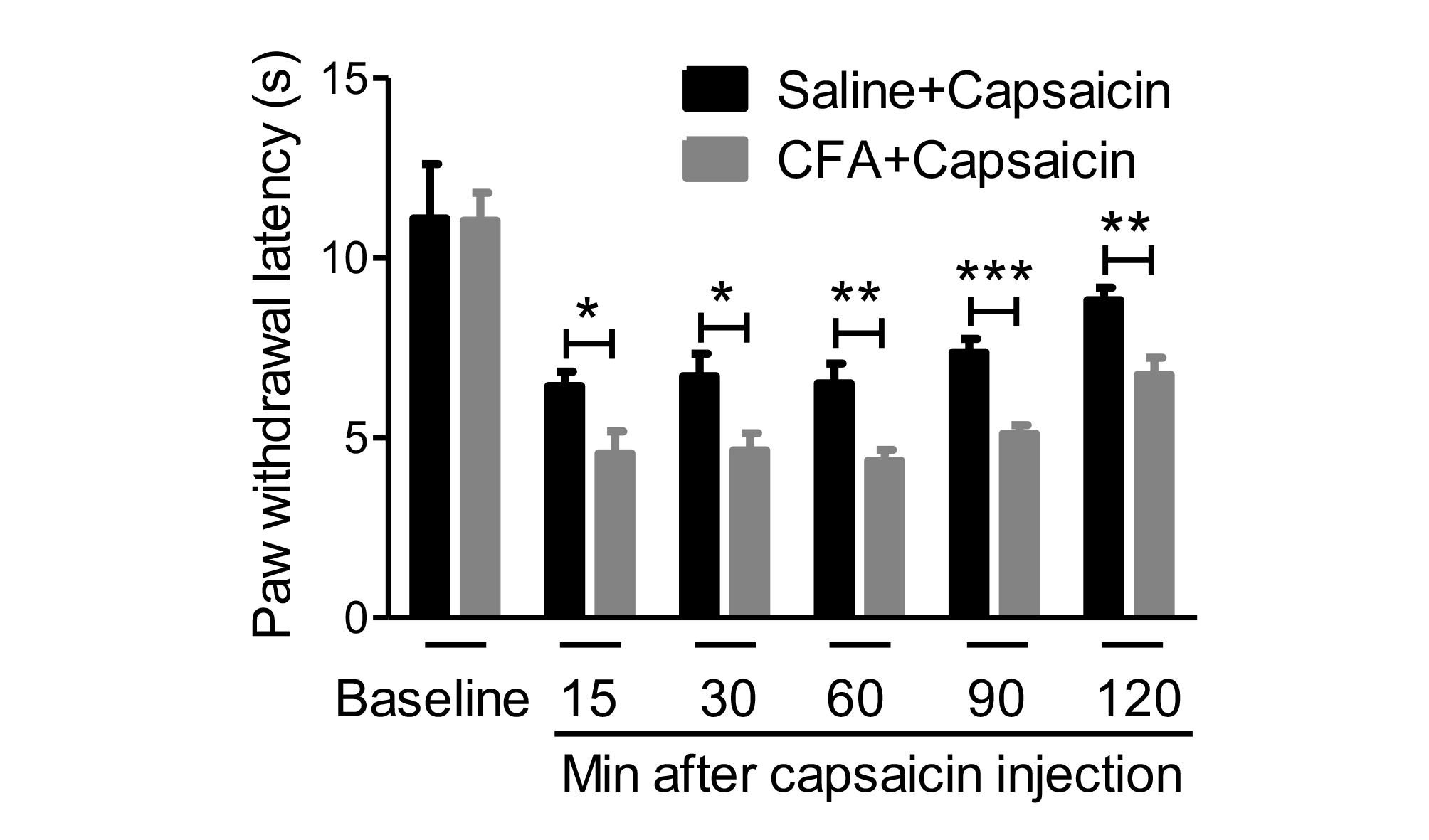

Supplement: FIGURE S2 — Aggravated capsaicin-induced pain in rats with chronic inflammatory pain experience. Rats with chronic inflammatory pain experience showed increased thermal hyperalgesia 15, 30, 60, 90 and 120 min after capsaicin injection. n = 8 in each group. p < 0.01, Saline+Capsaicin vs. CFA+Capsaicin, one-way ANOVA; *p < 0.05, **p < 0.01, ***p < 0.001, Saline+Capsaicin vs. CFA+Capsaicin, t test. [file Image_2.TIF]

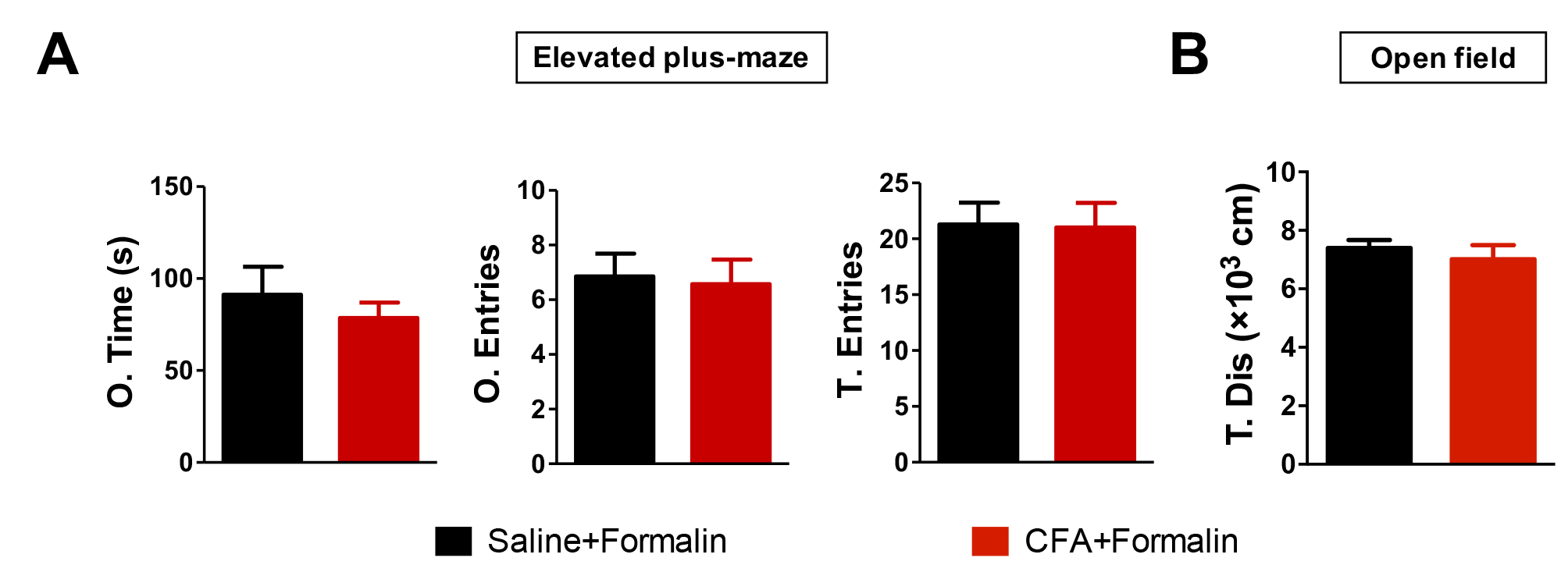

Supplement: FIGURE S3 — Similar levels of anxiety-like behaviors after formalin injection between CFA chronic pain group and saline control group. (A) Rats with chronic pain experience showed similar levels of anxiety-like behaviors in the elevated plus-maze test after formalin injection, indicated by similar time spent (O.Time, left) and entries (O.Entries, middle) into the open arms between groups. Total arm entries (T.Entries, right) were similar either. n = 8 in each group. CFA+Formalin vs. Saline+Formalin, t test. (B) Similar levels of locomotion after formalin injection between CFA and saline groups, indicated by similar total distance traveled (T.Dis) in the field. n = 8 in each group. CFA+Formalin vs. Saline+Formalin, t test. [file Image_3.TIF]

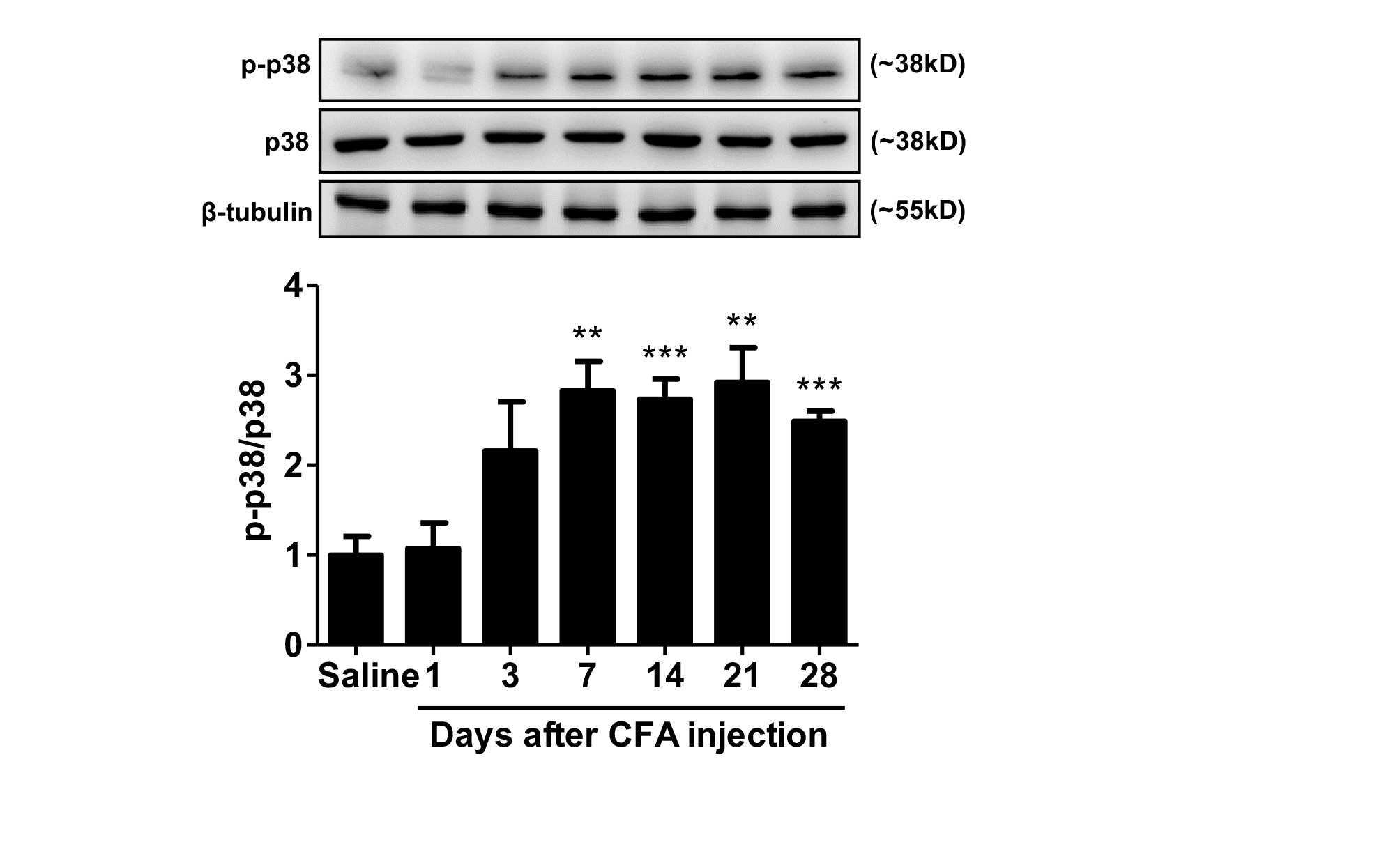

Supplement: FIGURE S4 — Increased levels of p-p38 in the PL after CFA injection. p-p38 in the PL increased in chronic pain rats 7 days after CFA injection, and maintained for at least 28 days. Representative Western blots of p-p38, p38 and β-tubulin were shown above the corresponding histogram. n = 5 in each group. ***p < 0.001, **p < 0.01, vs. Saline, one-way ANOVA. [file Image_4.tif]

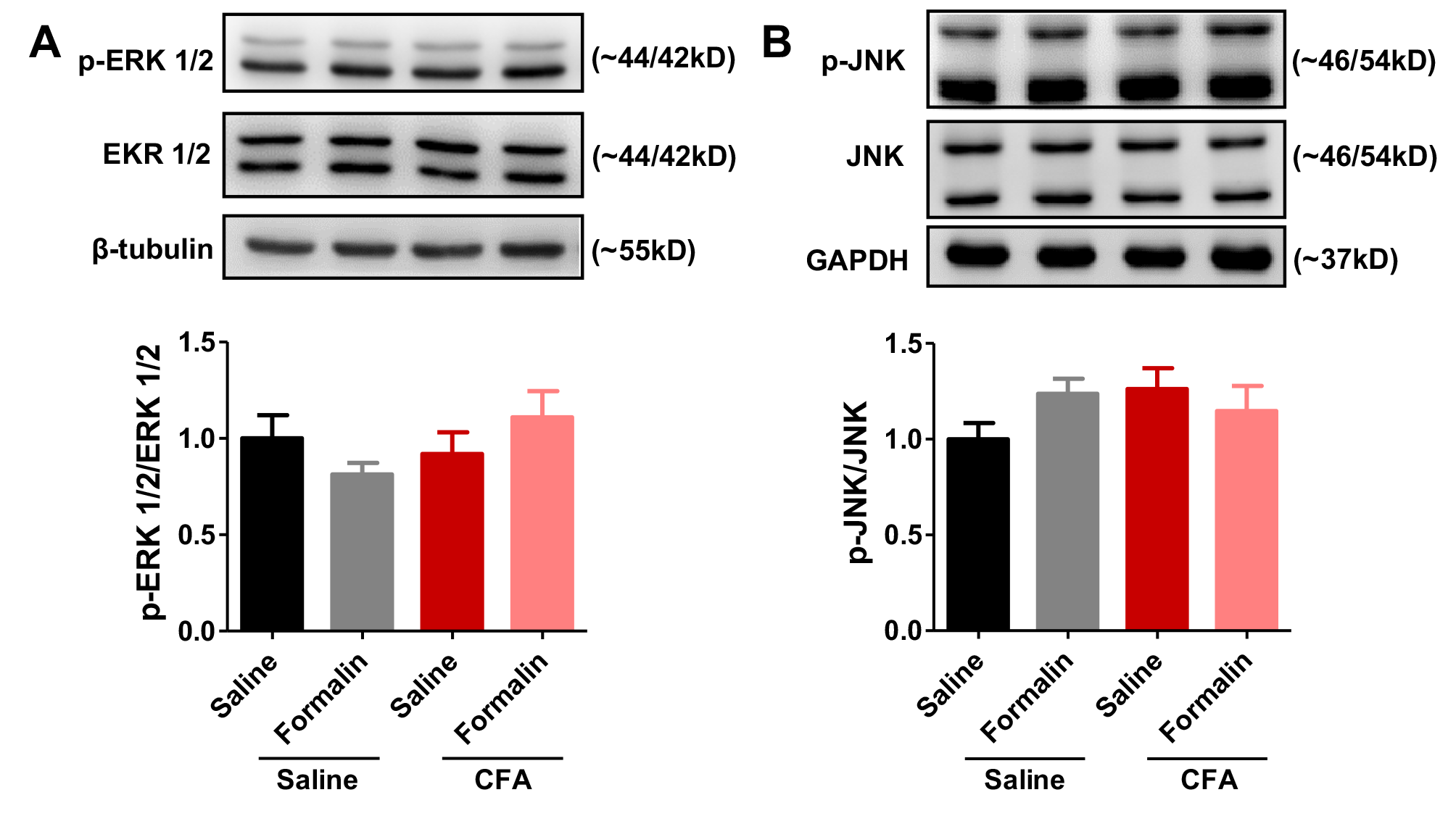

Supplement: FIGURE S5 — Chronic pain experience and formalin injection do not affect the phosphorylation of ERK1/2 and JNK in PL. (A) Similar levels of p-ERK1/2 in PL in rats with chronic pain experience. Formalin injection did not affect the phosphorylation of ERK1/2 in PL in rats with or without chronic pain experience. Representative Western blots of p-ERK1/2, ERK1/2 and β-tubulin were shown above the corresponding histogram. n = 8 in each group, one-way ANOVA. (B) Lack of significant changes of p-JNK in PL in rats with chronic pain experience. Formalin injection did not affect the phosphorylation of JNK in PL in rats with or without chronic pain experience. Representative Western blots of p-JNK, JNK and GAPDH were shown above the corresponding histogram. n = 8 in each group, one-way ANOVA. [file Image_5.tif]

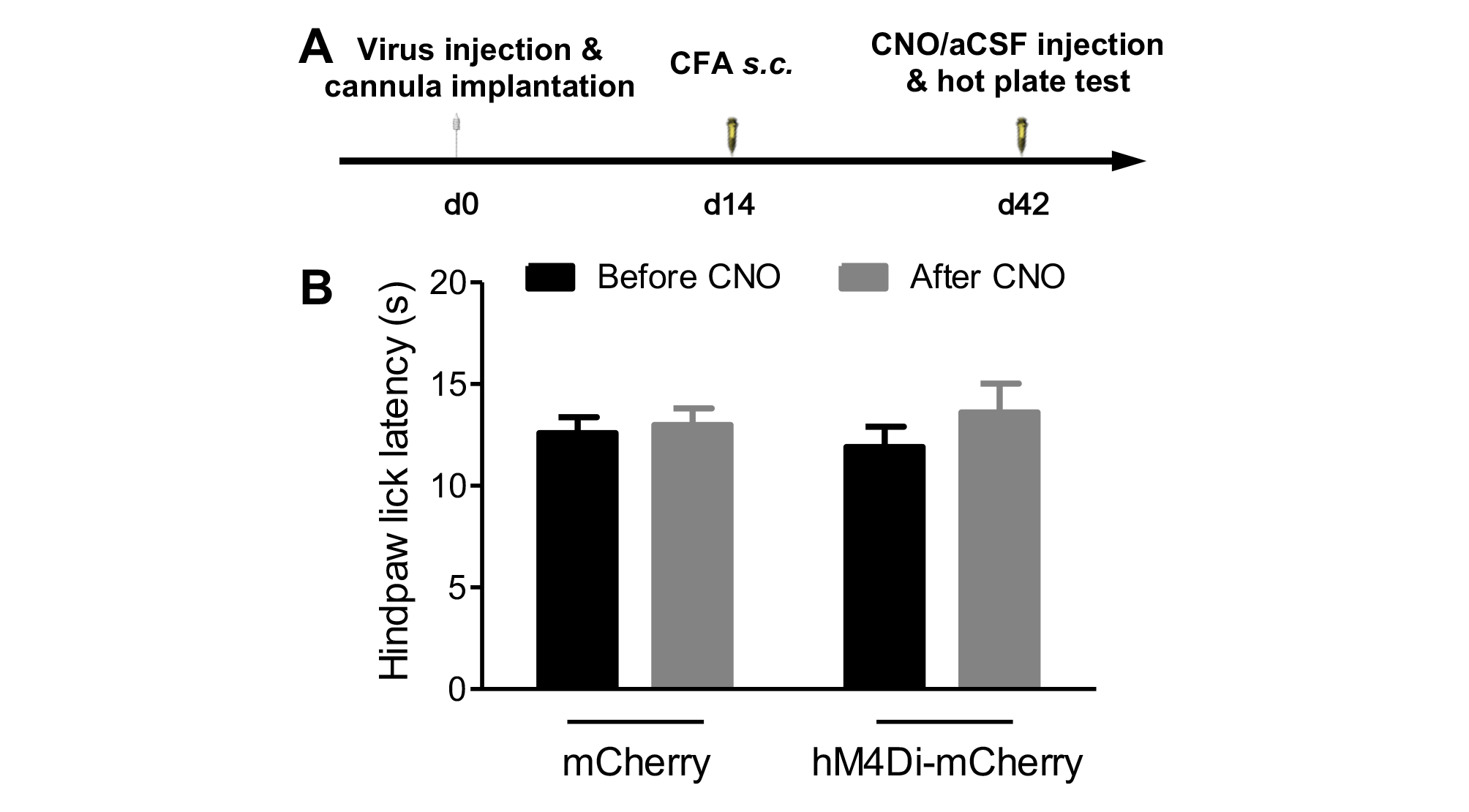

Supplement: FIGURE S6 — Inhibiting PL–PAG pathway does not affect physiological pain after chronic inflammatory pain recovery. (A) A diagram showing the time line of the experiment. The CNO/aCSF micro-injection and hot plate test were performed 28 days after CFA injection. Virus injection and cannula immplantation surgery were performed 14 days before CFA injection. (B) Compared to the control, inhibiting PL–PAG pathway did not affect physiological pain after chronic inflammatory pain recovery. n = 6 in each group. Before vs. After CNO injection, paired t test. [file Image_6.TIF]

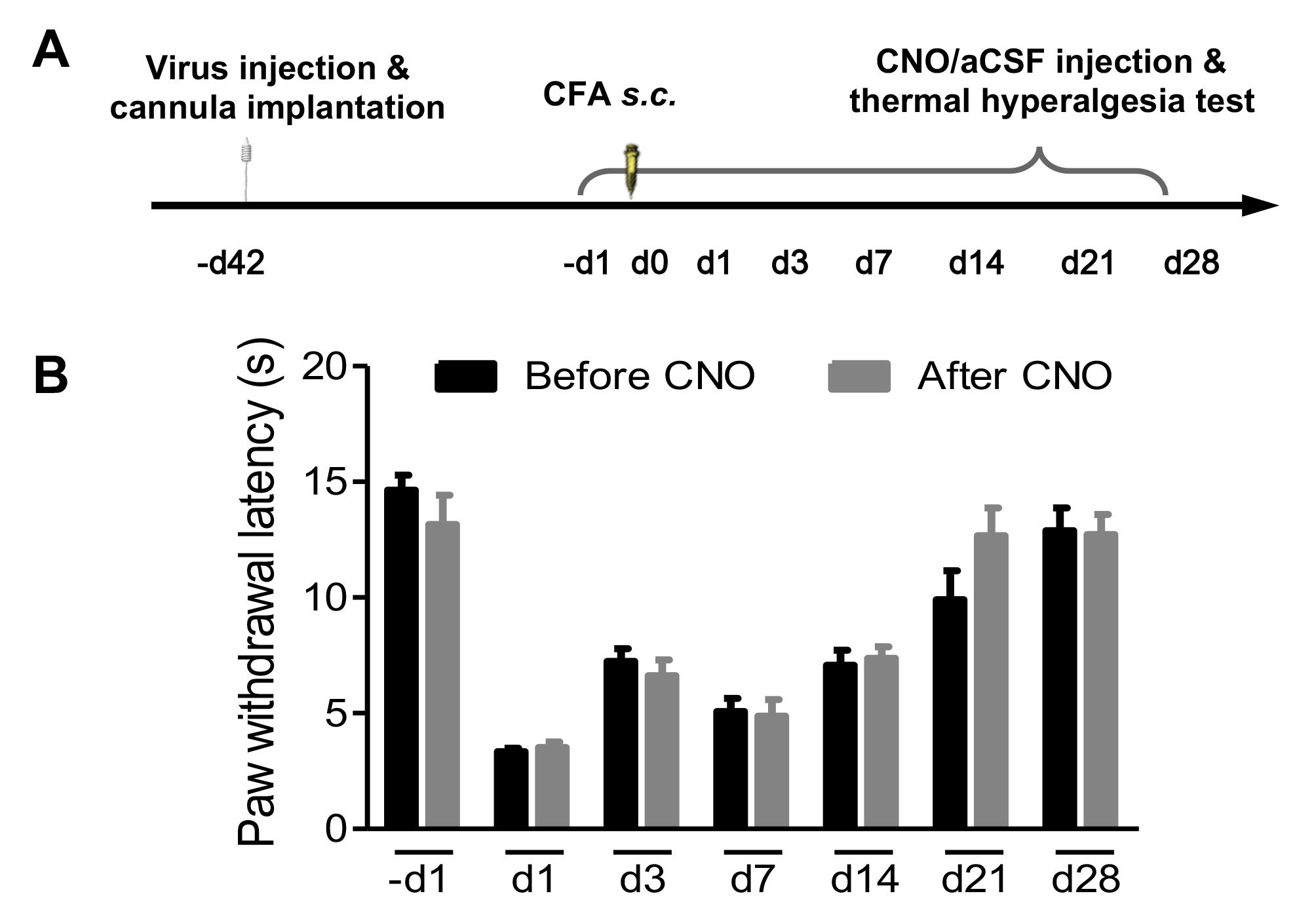

Supplement: FIGURE S7 — Inhibiting PL–PAG pathway does not affect thermal hyperalgesia in the development of CFA-induced chronic inflammatory pain. (A) A diagram showing the experiment time line. CNO/aCSF micro-injection and thermal hyperalgesia test were performed 1 day before and 1, 3, 7, 14, 21 and 28 days after CFA injection. Virus injection and cannula implantation surgery were performed 6 weeks before CFA injection. (B) Compared to the control, inhibiting PL–PAG pathway did not influence thermal hyperalgesia in the development of CFA-induced chronic inflammatory pain. n = 7 in each group. Before vs. After CNO injection, paired t test. [file Image_7.TIF]

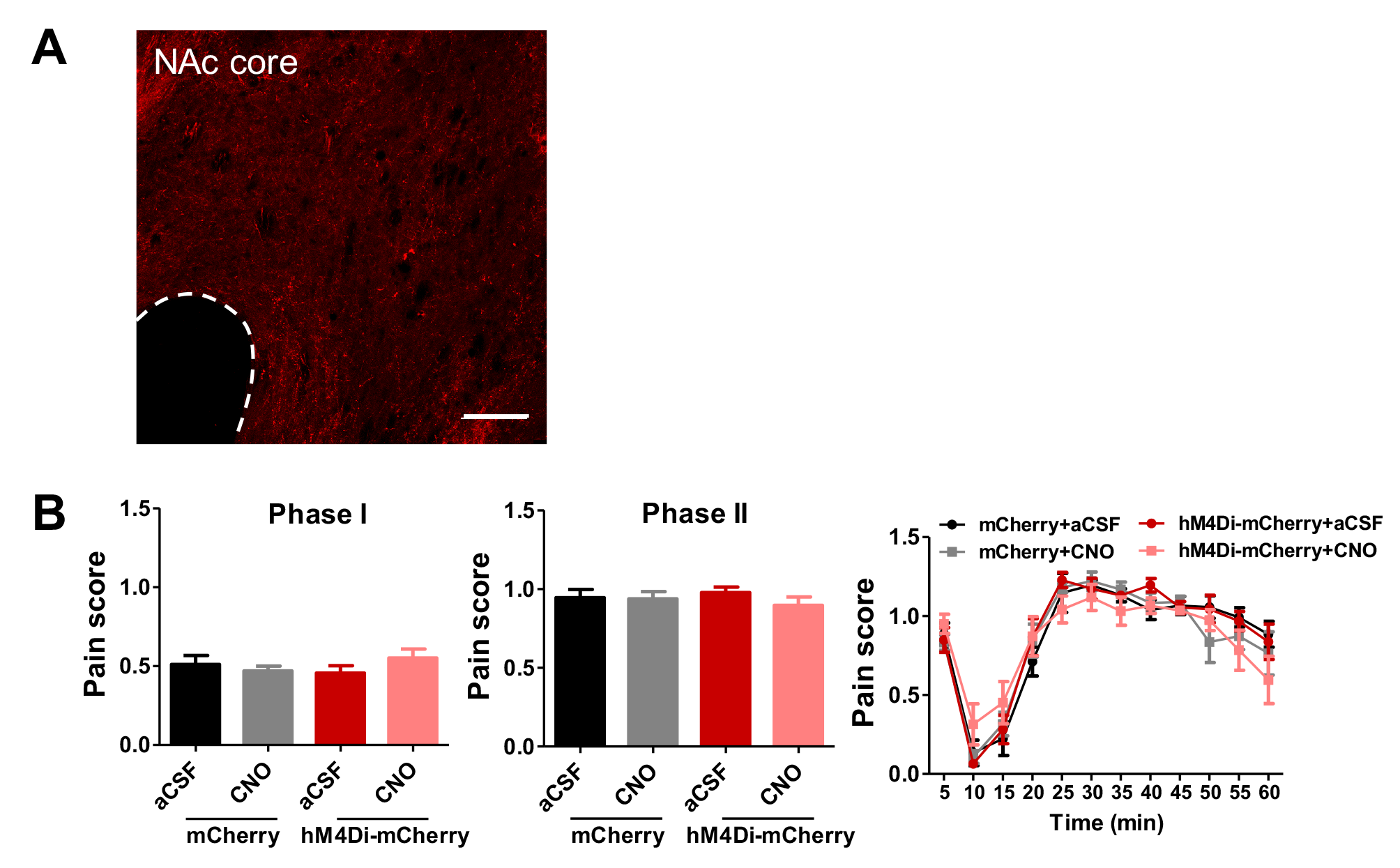

Supplement: FIGURE S8 — PL–NAc core pathway does not affect aggravated formalin pain in rats with chronic pain experience. (A) Confirmation of AAV5-CaMKIIα-hM4D(Gi)-mCherry virus expression in PL projection in NAc core (red). Scale bars: 100 μm. (B) Inhibiting PL–NAc core pathway did not relieve the aggravated formalin pain in either phase I (the left column) or phase II (the middle column) in CFA group. n = 8 in each group, one-way ANOVA. Detailed pain scores were shown in every 5 min (the right column). n = 8 in each group, ANOVA with repeated measures and Bonferroni post hoc test. [file Image_8.TIF]
